# Urinary Incontinence and Sleep Quality in Older Women with Type 2 Diabetes: A Cross-Sectional Study

**DOI:** 10.3390/ijerph192315642

**Published:** 2022-11-24

**Authors:** Chia-Hui Li, Min-Huey Chung, Chun-Hou Liao, Ching-Chieh Su, Yen-Kuang Lin, Yuan-Mei Liao

**Affiliations:** 1 School of Nursing, College of Nursing, Taipei Medical University, Taipei City 110, Taiwan, Republic of China (R.O.C.);; 2 Department of Surgery, Cardinal Tien Hospital, New Taipei City 231, Taiwan, Republic of China (R.O.C.);; 3 Department of Urology, Cardinal Tien Hospital, New Taipei City 231, Taiwan, Republic of China (R.O.C.); 4 College of Medicine, Fu Jen Catholic University, New Taipei City 242, Taiwan, Republic of China (R.O.C.); 5 Department of Endocrinology, Cardinal Tien Hospital, New Taipei City 231, Taiwan, Republic of China (R.O.C.);; 6 Dr. Su Diabetes Clinic, New Taipei City 231, Taiwan, Republic of China (R.O.C.); 7 Graduate Institute of Athletics and Coaching Science, National Taiwan Sport University, Taoyuan 333, Taiwan, Republic of China (R.O.C.);; 8 Institute of Clinical Nursing, College of Nursing, National Yang Ming Chiao Tung University, Taipei City 112, Taiwan, Republic of China (R.O.C.)

**Keywords:** body mass index, diabetes, night-time voiding frequency, quality of life, sleep quality, urinary incontinence

## Abstract

Background: Urinary incontinence (UI) and poor sleep negatively affect health-related quality of life (HRQoL). This study explored the UI-related factors and the relationships between UI, sleep quality, and HRQoL. Methods: This cross-sectional study collected data from 237 women with type 2 diabetes. Multivariate logistic regression was conducted to identify the factors associated with UI. One-way analysis of variance was used to compare the mean sleep quality and HRQoL scores of women without UI and those who experienced UI of varying severities. Correlation coefficients were estimated, and multivariate linear regression was conducted to examine the relationships between UI severity, sleep quality, and HRQoL. Results: Of the 237 women, 115 (48.52%) experienced UI and 139 (58.65%) were poor sleepers. The three factors associated with UI were advanced age, a higher body mass index, and a history of vaginal delivery. Significant associations between UI severity and sleep quality and between sleep quality and HRQoL were revealed. UI severity and night-time voiding frequency were both associated with sleep quality. Conclusions: One factor associated with UI (body mass index) is modifiable. UI severity is associated with sleep quality as the possible influence of night-time voiding frequency on sleep quality has been considered.

## 1. Introduction

Urinary incontinence (UI) refers to involuntary urine leakage. Stress UI is women’s most common type of UI, occurring during effort, exertion, sneezing, or coughing. The other two common types are urge UI, which occurs accompanied by or immediately preceded by urgency, and mixed UI, which is associated with urgency, effort, exertion, sneezing, or coughing [1]. More than one-third of older women experience UI. The prevalence of all types of UI significantly increases in women aged ≥60 years [2,3,4,5], and it is higher in women with diabetes than in women without diabetes [2,3,4,5,6]. Data obtained from general Asian middle-aged and older women showed that 45–55% of women experienced any type of UI; stress (20–30%) and mixed UI (15–20%) were more prevalent than urge UI (<10%) [4,5]. A study investigating lower urinary tract symptoms (LUTS) among women with type 2 diabetes showed that stress (29%) and urge UI (22%) were common; however, a prevalence of mixed UI was not reported [7]. Women experiencing UI may not report it to healthcare providers, although it often interferes with their daily tasks and activities. Unrelieved UI may negatively influence women’s physical health, mental health, and health-related quality of life (HRQoL) [8,9,10].

Factors associated with all types of female UI may include advanced age, obesity or high body mass index (BMI), certain gynecological (e.g., high parity, vaginal delivery, hysterectomy), medical (e.g., diabetes, hypertension) or psychological conditions, and some personal habits (e.g., caffeine consumption, constipation, low physical activity level) [2,3,5,6,10,11]. A study conducted among Chinese women showed that high parity, obesity, and diabetes are associated with stress and mixed UI [5]. Limited studies on women with diabetes revealed that factors associated with UI included higher HbA1C, higher BMI, use of hormone replacement therapy, smoking habits, and low physical activity levels [12,13]. For the optimal management of UI, healthcare providers should pay special attention to modifiable factors associated with UI [14,15].

Poor sleep is also common among older women and women with type 2 diabetes [16,17,18,19]. Maintaining adequate sleep can contribute to optimal health and HRQoL for older women [20], and optimal glycemic control for individuals with diabetes [21]. Older women with concurrent UI and poor sleep quality have poor HRQoL [22]. Moreno et al. [17] reported significant associations between poor sleep and both UI and night-time voiding frequency in older adults aged ≥60 years. Recent studies on adults without diabetes have shown an essential relationship between UI and poor sleep: (a) women with more episodes of UI experienced poorer sleep quality compared to women with fewer episodes of UI; (b) women with more frequent UI episodes or a higher level of urine leakage amount (e.g., enough to soak a pad) had poorer sleep quality compared to those with lower UI severity [10,20]; and (c) community dwelling older adults with UI were more likely to suffer from poor sleep than those without UI [23].

UI and poor sleep are common among older women and women with type 2 diabetes [3,4,6,16,17,18,19]. Further, UI and poor sleep both negatively affect women’s health and HRQoL [8,9,10,20,22]. Longitudinal studies have shown a close relationship between LUTS and poor sleep [24,25], and between UI and poor HRQoL [26]. Further, poor sleep and low HRQoL may impede optimal glycemic control in individuals with diabetes [21,27]. As women with diabetes may concurrently experience UI and increased night-time voiding frequency [7,13], healthcare providers should pay attention to both UI and night-time voiding frequency because both of them are associated with poor sleep [17]. Compared to the amount of studies examining the relationship between night-time voiding frequency and sleep, studies examining the relationship between UI and sleep were relatively fewer, and they were mainly conducted in individuals without diabetes [10,20,23]. Limited studies have explored the possible relationships and negative influences of UI and poor sleep among older women with type 2 diabetes. Thus, more studies should be conducted on this specific population to examine the relationships between UI, sleep quality, and HRQoL.

The present study explored factors associated with UI and the relationships between UI, sleep quality, and HRQoL in a sample of older women with type 2 diabetes. Research is required to identify UI-related factors and obtain better knowledge regarding the relationship between UI, sleep quality, and HRQoL. Our findings would assist in the development of adequate interventions to reduce UI and overcome its negative influences, and to promote this population’s sleep, overall health, and HRQoL.

## 2. Materials and Methods

### 2.1. Study Design and Ethical Consideration

This cross-sectional study obtained ethical approval from the Institutional Review Board of the Cardinal Tien Hospital (110-3-1-011) before the start of the study.

### 2.2. Setting and Sample

The study site was an endocrinology outpatient department at a hospital located in northern Taiwan. Data were collected from a convenience sample of 237 women aged 60–79 years who were diagnosed with type 2 diabetes for more than six months and had intact cognition and communication abilities. Exclusion criteria were as follows: physical impairment; taking antipsychotic medications; having a history of spinal surgery or cardiovascular, renal, or nervous system diseases; and having experienced urinary tract infection in the past month. One primary investigator (Li CH) reviewed the information in women’s medical records to screen for age, duration of type 2 diabetes, major cognitive impairments, use of psychiatric medications, history of surgery and diseases, or a urinary tract infection in the past month. This primary investigator further screened for physical impairment and intact communication ability among those preliminarily determined eligible participants in the outpatient departments.

We performed a post hoc power analysis via G-Power 3.1.9.4 (Heinrich Hiene University, Dusseldorf, Germany) to estimate the obtained power using logistic regression. A history of vaginal delivery, which is a common factor associated with female UI [3,6,11], was used to conduct the power estimation. The odds of experiencing UI increased with a history of vaginal delivery, and the odds ratio obtained from our study (OR = 3.147) was used to conduct the power estimation. A power of >0.80 is generally considered acceptable [28]. This study has an adequate power of 0.92 under the following assumptions: a 2-tailed α of 0.05, a sample size of 237, 48.52% of the study participants experienced UI, and 79.32% of the study participants had a history of vaginal delivery.

### 2.3. Instrument

A questionnaire was used to collect information related to individual characteristics, LUTS, sleep quality, and HRQoL. Information about individual characteristics was obtained using investigator’s self-developed items comprising women’s age, body height/weight, parity (number of times given birth) and vaginal delivery history, time since menopause, use of hormone replacement therapy, experience of chronic constipation, smoking habit, habits of regular exercise, pelvic floor muscle exercise, caffeine/alcohol consumption, and medical-related conditions (e.g., histories of hysterectomy, hypertension, time since diagnosis of diabetes, and recent HbA1C levels). We calculated the BMI by dividing a woman’s weight (kg) by the square of her body height (m^2^). Women with a BMI ≥ 27.00 kg/m^2^ were defined as obese [29]. Regular exercise was defined as performing physical activity ≥3 times per week for ≥30 min that is vigorous enough to result in sweating [30].

Information related to LUTS, including UI and night-time voiding frequency, was collected using the LUTS subscale of the Taiwan Teacher Bladder Survey [31]. There are three groups of LUTS: storage (e.g., UI, nocturia, increased daytime frequency, and urgency), voiding (e.g., a slow stream, an intermittent stream, and hesitancy), and post-micturition (e.g., a feeling of incomplete emptying) symptoms. Definitions of LUTS were based on the International Continence Society standardization report [1]. Approval was obtained to use the LUTS subscale for measuring 8 common female LUTS in this study. The internal consistency of the LUTS subscale (Kuder–Richardson formula, 21 = 0.71), content validity (content validity index = 1.00), and test–retest reliability (Phi correlations = 0.74–1.00) of the subscale items were adequate. Women were asked to respond to each urinary symptom according to their personal experiences over the past month. The presence of UI and the other LUTS was confirmed if the symptoms lasted more than one month and occurred at least once a month. Information related to night-time voiding frequency was collected by the question: “How many times did you typically get up at night to urinate?” If a woman had experienced UI, she was asked to report the duration and frequency of UI, amount of urine leakage, and situations when UI occurred [31]. Sandvik’s severity index, computed by multiplying the self-reported frequency (4 levels) by the amount of urine leakage (3 levels), was used to represent the severity of UI. The resulting Sandvik severity index values (1-12) were categorized into four levels: 1–2 = slight, 3–6 = moderate, 8–9 = severe, and 12 = very severe. Sandvik et al. obtained a fair and significant correlation coefficient value (0.54, *p* < 0.01) between the four-level severity index values and pad-weighting test results by analyzing the data obtained from 265 women [32].

The Pittsburgh Sleep Quality Index (PSQI) was used to measure women’s sleep quality over the past month [33]. Approval was obtained to use the Chinese version of the PSQI (CPSQI) in this study [30]. The PSQI/CPSQI global score (range = 0–21) was generated by summing the seven component scores (range = 0–3): subjective overall sleep quality, sleep latency, sleep duration, habitual sleep efficiency, sleep disturbances, use of sleeping medication, and daytime dysfunction. A higher PSQI or CPSQI global or component score indicates poorer sleep quality [33,34]. The CPSQI has acceptable test–retest reliability (0.77–0.85) and an overall reliability coefficient of 0.82–0.83. A test of known-group validity revealed that the CPSQI could adequately discriminate between poor sleepers and those who slept enough. A woman with a CPSQI global score of >6 was defined as a poor sleeper [34].

The Short Form 36 (SF-36) Health Survey was used to assess HRQoL. It included an item relating to self-perceived changes in health and 35 items belonging to eight HRQoL aspects (physical functioning, role limitation due to physical health problems, bodily pain, general health, vitality, social functioning, role limitation due to emotional problems, and mental health). The physical and mental component summary (PCS and MCS) HRQoL scores can be calculated using the eight-aspect HRQoL scores and relevant information in the user’s manual. A higher PCS and MCS HRQoL score indicates a superior combination of physical or mental functions [35,36]. The SF-36 Health Survey, Taiwan version, has adequate psychometric properties comprising ideal internal consistency reliability (Cronbach’s α > 0.70) and satisfactory construct validity confirmed by multi-trait scaling analyses, principal component analysis, and a known group validity test [37].

### 2.4. Study Procedure

The study procedure, data collection, and risks or benefits were explained to the eligible participants. All participants provided written consent before data collection. Two data collectors trained by the principal investigator interviewed women in the outpatient department and recorded their responses to the questionnaire (20–25 min). An incentive gift (USD ≈ 7) was provided to each participant.

### 2.5. Data Analysis

SPSS 21.0 software (SPSS, Chicago, IL, USA) for Windows was used for data analyses. Multivariate logistic regression analysis was performed to identify factors associated with UI. The variable selection procedure was based on the process proposed by Hosmer et al. [38]: variables with *p* values < 0.20 obtained from independent *t* test for continuous variables or Chi-squared test for categorical variables were selected for the first regression model. For the significant factors associated with UI, we present estimated odds ratios (ORs) and associated 95% confidence intervals (CIs).

To examine the relationship between UI, sleep quality, and HRQoL, we categorized participants into three groups: those without UI, with slight to moderately severe UI, and with severe to very severe UI. Further, we compared the mean sleep quality and PCS/MCS HRQoL scores of the three groups using a one-way analysis of variance (ANOVA). Using the information obtained from the 115 women with UI, we estimated Spearman’s correlation coefficients between UI severity or night-time voiding frequency with sleep quality and PCS/MCS HRQoL. We conducted multivariate linear regression analysis with sleep quality as the dependent variable and UI severity and night-time voiding frequency as the independent variables. Night-time voiding frequency was included in the estimation of correlation coefficients and linear regression analysis because of its possible association with sleep quality [15]. Age and BMI were included in the linear regression analysis as covariates as they are reported to be potential correlates of poor sleep quality in women [14,16].

## 3. Results

Table 1 presents the participants’ individual characteristics. Of the 237 women, 115 (48.52%) experienced UI, 45 (18.99%) had night-time voiding frequency ≥3 episodes, and 139 (58.65%) were poor sleepers (CPSQI global score >6). Of the 115 women with UI, 20 (17.39%) had night-time voiding frequency ≥3 episodes, 73 (63.48%) were poor sleepers, and only 8 (6.96%) reported UI to their healthcare providers. Table 2 presents symptom-related conditions of the 115 women with UI. Of the 115 women with UI, 69 (60%) experienced moderate to very severe UI and most experienced stress (*n* = 62, 53.91%) or mixed UI (*n* = 49, 42.61%) (Table 2).

### 3.1. Factors Associated with UI

The first multivariate logistic regression model included four candidate factors associated with UI. The mean age (*p* = 0.024) and mean BMI (*p* < 0.001) of women with UI were significantly higher than those of women without UI. Chi-squared tests revealed that women who had given birth ≥3 times (*p* = 0.029) or had a history of vaginal delivery (*p* = 0.001) were more likely to experience UI than those who did not have these characteristics (Table 1). Multivariate logistic regression analysis identified three significant factors associated with UI. The odds of experiencing UI increased with advanced age (OR = 1.057, 95% CI = 1.001, 1.116; *p* = 0.049), higher BMI (OR = 1.116, 95% CI = 1.042, 1.196; *p* = 0.002), and history of vaginal delivery (OR = 3.147, 95% CI = 1.508, 6.568; *p* = 0.002).

### 3.2. Relationship between UI, Sleep Quality, and HRQoL

One-way ANOVA used the data obtained from 237 women. The CPSQI global score and scores of the four components (subjective overall sleep quality, sleep disturbances, use of sleeping medication, and daytime dysfunction) for women with severe to very severe UI were significantly higher than those for women without UI or with slight to moderate UI severity. The MCS HRQoL score for women with severe to very severe UI was significantly lower than that for women without UI (Table 3).

In the 115 women with UI, significant correlation coefficients were obtained between UI severity and sleep quality (*r_s_* = 0.40, *p* < 0.001) and MCS HRQoL (*r_s_* = −0.23, *p* = 0.014), between night-time voiding frequency and sleep quality (*r_s_* = 0.43, *p* < 0.001), and between sleep quality and PCS (*r_s_* = −0.21, *p* = 0.022) and MCS (*r_s_* = −0.35 *p* < 0.001) HRQoL. Multivariate linear regression analysis adjusted for age and BMI showed that both UI severity and night-time voiding frequency were significantly associated with sleep quality and explained 23.4% of the total variance (Table 4).

## 4. Discussion

Our study results support previous findings regarding the high prevalence of UI in older women and women with type 2 diabetes [2,3,4,5,6]. Studies have shown that obesity is associated with poor sleep quality in middle-aged and older women [16,39]. Beyond the possible influence associated with urinary symptoms, the high proportion of poor sleep quality (>58%) in this study may also be associated with the high prevalence of obesity (37.98%) of our participants.

### 4.1. Factors Associated with UI

Our findings regarding age, BMI, and a history of vaginal delivery being significant factors associated with UI are consistent with prior findings [3,6,11]. Aging and menopause are significantly associated with female UI. For most women, neuromuscular changes in the pelvic floor muscles associated with aging could result in decreased overall muscle strength and power [40]. Estrogen receptors are expressed throughout the lower urinary tract, particularly in the structures related to urinary continence (e.g., the urethra, bladder trigone, and pelvic floor muscles and ligaments). Older women experiencing a combination effect of aging and estrogen withdrawal will experience anatomic, physiologic, and functional changes in the lower urinary tract, which may lead to UI [40,41].

A majority of our participants had a history of vaginal delivery. It has been suggested that vaginal delivery and high parity are associated with UI, possibly because of injuries to pelvic floor muscles, nerves, and connective tissues [11,42]. A woman’s pelvic floor structure, strength of the soft tissue, process/mechanism of delivery, and size of delivered baby can also affect injuries resulting from childbirth [43]. To better understand the possible effects of the delivery process and pelvic floor function on UI, obtaining more detailed information about these conditions in further research is recommended.

Healthcare providers should pay special attention to BMI because BMI is modifiable [14,40,44]. The relationship between higher BMI and UI may be explained by the mechanical effects of higher BMI on intra-abdominal pressure, intravesical pressure, and urethral mobility. Increased intra-abdominal pressure may lead to weakening of the pelvic floor innervation and musculature, and an increase in intravesical pressure and urethral mobility [40,45]. More than half (~54%) of the middle-aged or older Taiwanese women were overweight (~29%; BMI 24.00–<27.00 kg/m^2^) or obese (~25%; BMI ≥27.00 kg/m^2^) [46]. There is evidence that behavioral weight loss interventions have beneficial effects on reducing UI in overweight and obese women [47,48]. Healthcare providers should focus on promoting the prevention of weight gain and losing excess body weight and developing continence management strategies for older women. Related interventions may include regular physical exercise and a healthy diet for maintaining a normal body weight and healthy BMI [14,15,30,40]. Conservative management of UI suitable for older women, such as maintaining good bowel movement habits, voiding pattern modifications, training the bladder, training the pelvic floor muscles, and urge-suppression techniques, can also be practiced [14,15,40].

Diabetes-related bladder dysfunctions including LUTS are difficult to study as individuals with diabetes are diverse. A more recent pathophysiology approach related to diabetes-related bladder dysfunctions believes that the dysfunctions involve a complex interaction of many cellular changes resulting from hyperglycemia [13]. Our finding regarding HbA1C being a non-significant factor associated with UI is different from prior study finding [12], although the mean HbA1C level of our participants with UI is higher than that of those without UI (*t* = 0.928, *p* = 0.354; 8.07 vs.7.87). A study with a large sample size tends to have a population with more diverse individual characteristics. The different findings revealed in our study (*n* = 237) and Wang et al.’s study (*n* = 7270) [12] may be explained by the different sample sizes involved in the two studies.

Management options for UI range from lifestyle modifications to invasive surgical interventions. First-line interventions include lifestyle, behavioral, and physical therapies. For example, pelvic floor muscle training, maintaining ideal body weight, and good bowel movement habits are beneficial for relieving stress, urge, and mixed UI. Further, management options suitable for mixed UI may include weight loss, timed voiding, bladder retraining, avoiding bladder irritants, and adjusting fluid intake arrangement [14,15]. Most of the 115 women with UI in our study experienced stress or mixed UI, and >70% (*n* = 84) experienced UI with slight to moderate severity levels. This result suggests that most women are suitable candidates for conservative management, such as pelvic floor muscle training or bladder training, to reduce their symptoms [14,15]. Pharmacological agents (i.e., anticholinergic drugs for urge UI, and serotonin–noradrenaline reuptake inhibitors for stress UI) or invasive/surgical interventions are available when the effects of first-line interventions are unsatisfactory [48]. However, a network meta-analysis by Balk et al. revealed that behavioral therapy, alone or combined with other interventions, is generally more effective than pharmacologic therapies alone in treating both stress and urge UI [49]. Healthcare providers can explain the benefits and possible limitations of different approaches, help women in choosing suitable approaches meeting their own needs, and periodically re-evaluate women’s management goals and preferences because these conditions may change with UI severity or time.

### 4.2. Relationship between UI, Sleep Quality, and HRQoL

To investigate these relationships, the sample of women with diabetes in the present study differed from that used in prior studies [10,20,22,23,26]. The adverse effects of poor sleep and HRQoL on optimal glycemic control among individuals with diabetes [21,27] support the importance of conducting the current study. Our study results regarding the relationship between UI and sleep quality support recent findings that women with more severe UI had poorer sleep quality [10,20,50]. Winkelman et al. reported that an increase in total daily UI episodes, total daily urgency UI episodes, and moderate to severe urge sensations were all associated with poorer sleep (*p* < 0.01). Possible reasons for the relationship between UI and sleep quality may include: (a) women with severe or very severe UI may experience social isolation or poor mental health, which may subsequently lead to poor sleep quality; (b) concerns about urine leakage during sleep may have negative influences on sleep; and (c) wet underpants or clothes may interfere with sleep. A recent 10-year longitudinal study showed that UI is associated with poor HRQoL and that UI may be a risk factor for poor HRQoL [26]. Our participants with high BMI, more severe UI, and poor sleep were associated with poor HRQoL, which is consistent with the findings revealed in Avis et al.’s longitudinal study [22]. Prior study findings [10,20,22,26,50], together with our results showing close relationships between UI with sleep quality and HRQoL, support that healthcare providers should treat UI as an essential issue among older women with diabetes.

Longitudinal studies have shown that poor sleep quality is significantly associated with the worsening of LUTS [24]. Araujo et al. [24] found that BMI played a role in the relationship between poor sleep quality and the development of UI or LUTS. Koolhaas et al. [51] proposed that the relationship between sleep and BMI may be bidirectional later in life. The complex associations between UI, BMI, and sleep quality have been revealed in previous longitudinal studies [24,25,51]. The high proportion of our study participants with UI and poor sleep warrants that healthcare providers should screen this population for UI and poor sleep, particularly those who are overweight or obese. Furthermore, healthcare providers should emphasize the critical associations between UI, BMI, and poor sleep when providing urological healthcare to this population.

In middle-aged adults with an overactive bladder, Ge et al. reported a significant correlation (*r_s_* = 0.32, *p* = 0.015) between UI severity and sleep disturbance, adjusting for age, gender, and night-time voiding frequency [50]. Moreno et al. found that both UI and night-time voiding are associated with poor sleep in older adults [17]. Winkelman et al. found significant relationships between poor sleep and increasing urgency of UI episodes per night and night-time voiding frequency [10]. Among women with type 2 diabetes, we found that not only is night-time voiding frequency associated with sleep quality, but also UI severity is associated with sleep quality. Healthcare providers should not assume that poor sleep in older women is primarily related to night-time voiding frequency, but should be aware that poor sleep may be associated with UI.

Healthcare providers should encourage women to report their symptoms and promote conservative management, which is likely to benefit this population. Insomnia-related behavioral treatments have beneficial effects not only on poor sleep, but also on night-time voiding frequency [52]. In addition to the management techniques suitable for managing UI or obesity presented in the previous section, interventions such as regular physical exercise, decreasing or avoiding caffeine consumption and alcohol intake, restricting fluid intake for several hours before sleep, implementing feasible modifications in sleeping environments, and maintaining sleep hygiene can be considered to reduce night-time voiding frequency and improve sleep [52,53,54].

We also found a close association between sleep quality and HRQoL, which is consistent with previously reported findings [10,20]. Given the known adverse effects of poor sleep on health and HRQoL, healthcare providers should ask women with UI about their sleep quality and HRQoL to deliver appropriate and timely management. Considering the adverse effects of UI and poor sleep on women’s physical health, mental health, and HRQoL [8,9,10], the vicious circle between LUTS and poor sleep [24,25], and the bidirectional relationship between poor sleep and obesity [51], healthcare providers should focus on simultaneously reducing UI and night-time voiding frequency, promoting a healthy BMI, and improving sleep to achieve women’s optimal health and HRQoL.

Future studies may consider using objective examinations (e.g., urodynamics and actigraphy) to validate the self-reported measures. Researchers can obtain information from a voiding diary, an urodynamics study, or a sleep laboratory to further investigate the underlying etiologies of UI, night-time voiding frequency, and poor sleep, and provide individualized management. Older adults with good sleep quality usually engage in sufficient physical activity [55]. We recommend using valid instruments (e.g., International Physical Activity Questionnaire) or devices (e.g., actigraph) to collect more precise data regarding physical activity levels in future studies. We also recommend conducting more longitudinal studies to examine how changes in UI severity or night-time voiding frequency are correlated with changes in sleep quality to enrich current knowledge about the relationship between UI, night-time voiding frequency, and sleep quality.

### 4.3. Limitation

This study has several limitations, including its cross-sectional design, which can only suggest an association, recall bias, self-reporting, and inability to evaluate the multiple correlates possibly contributing to UI in the participants. This study did not assess physical activity level or the use of medications to relieve poor sleep or neuropathic pain, which may affect sleep quality. Convenience sample and sample exclusion (e.g., physical impairment, and taking antipsychotic medications) may have reduced the generalizability of the study results.

## 5. Conclusions

One identified factor associated with UI, BMI, may be modifiable. Among women with UI, UI severity was significantly associated with sleep quality and MCS HRQoL, and sleep quality was closely related to PCS/MCS HRQoL. Higher levels of UI severity are associated with poorer sleep quality, given that the possible influence of night-time voiding frequency on sleep quality has been considered. This pioneer quantitative study concurrently investigated the possible influences of UI and night-time voiding frequency on sleep quality in older women with type 2 diabetes. This study extended the current understanding on the relationships between UI, sleep quality, and HRQoL among women with type 2 diabetes and may serve as a reference for clinical diabetes care and future research.

Healthcare providers need to pay attention to sleep quality and HRQoL when they provide health care to older women with type 2 diabetes, especially those experiencing UI, because poor sleep and low HRQoL may impede optimal glycemic control. Treating UI may improve poor sleep quality among women with both bothersome conditions. Healthcare providers should be alert to UI as it may often be unreported and therefore under-treated. The findings highlight the importance of educating women with diabetes and healthcare providers about the adverse effect of UI on sleep quality because UI’s adverse effect, compared to nocturia’s adverse effect, is more likely to be overlooked. Healthcare providers should screen for urinary symptoms (e.g., UI and nocturia) and poor sleep and offer appropriate beneficial behavioral interventions and non-pharmacological lifestyle management for UI, night-time voiding frequency, or poor sleep, which may promote healthy pelvic floor function, healthy BMI, bladder health, and adequate sleep quality, and potentially lead to optimal HRQoL.

## Figures and Tables

**Table 1:** Individual characteristics of study participants (*n* = 237).

Variable	All (*n* = 237)		With UI (*n* = 115)		Without UI (*n* = 122)		^†^*t* Test or Chi-Square Value	*p*
	*n*	%	*n*	%	*n*	%		
Age (years; 60–79)	M ± SD 65.59 ± 5.12		66.37 ± 5.60		64.85 ± 4.52		^†^ 2.28	0.024 *
60–64	113	47.68	49	42.60	64	52.46		
65–69	77	32.49	33	28.70	44	36.07		
70–79	47	19.83	33	28.70	14	11.48		
Body mass index (kg/m^2^; 17.98–43.00)	M ± SD 25.93 ± 4.27		26.92 ± 4.05		25.00 ± 4.28		^†^ 3.54	<0.001 ***
<24.00 kg/m^2^ (underweight or normal)	86	36.28	30	26.09	56	45.90		
24.00–<27.00 kg/m^2^ (overweight)	61	25.74	31	26.95	30	25.59		
≥27.00–<30.00 kg/m^2^ (slightly obese)	50	21.10	29	25.22	21	17.21		
≥30.00–43.00 kg/m^2^ (moderately or severely obese)	40	16.88	25	21.74	15	12.30		
Parity (number of times given birth)								
0	12	5.06	3	2.61	9	7.38	4.75	0.029 *
1	12	5.06	4	3.48	8	6.56	(<3 vs. ≥3 times)	
2	67	28.27	29	25.22	38	31.15		
3	75	31.65	32	27.82	43	35.25		
≥4	71	29.96	47	40.87	24	19.67		
Vaginal delivery history							11.96	0.001 **
No	49	20.68	13	11.30	36	29.51	(no vs. yes)	
Yes: Once	13	5.48	5	4.35	8	6.56		
Twice	48	20.25	25	21.74	23	18.85		
Three times	62	26.16	30	26.09	32	26.23		
≥4 times	65	27.43	42	36.52	23	18.85		
Time since menopause							1.57	0.210
>5–10 years	58	24.47	24	20.87	34	27.87	(≤10 vs. >10 years)	
>10 years	179	75.53	91	79.13				
Use of hormone replacement therapy							--	--
Yes	3	1.26	1	0.87	2	1.64		
Chronic constipation							0.74	0.388
Yes	60	25.32	32	27.83	28	22.95	(no vs. yes)	
Smoking habit							--	--
Yes	9	3.80	2	1.74	7	5.74		
Regular exercise							1.58	0.209
Yes	113	47.68	50	43.48	63	51.64	(no vs. yes)	
Pelvic floor muscle exercise							--	--
Yes	5	2.11	1	0.87	4	3.28		
Caffeine consumption							0.03	0.865
Yes	125	52.74	60	52.17	65	53.28	(no vs. yes)	
Alcohol consumption							0.51	0.476
Yes	12	5.06	7	6.09	5	4.10	(no vs. yes)	
Hysterectomy							--	--
Yes	5	2.11	4	3.48	1	0.82		
Hypertension							0.07	0.794
Yes	101	42.62	50	43.48	51	41.80	(no vs. yes)	
Time since diagnosis of type 2 diabetes							1.16	0.281
≤5 years	65	27.43	33	28.70	32	26.23	(≤10 vs. >10 years)	
>5 years–≤10 years	61	25.74	24	20.87	37	30.33		
>10 years	111	46.83	58	50.43	53	43.44		
HbA1C (%; 5.60–16.20)	M ± SD 7.97 ± 1.71		8.07 ± 1.90		7.87 ± 1.50		^†^ 0.93	0.354
<7%	66	27.85	34	29.56	32	26.23	0.33	0.567
7.0%–<8.0%	83	35.02	33	28.70	50	40.99	(<7% vs. ≥7%)	
8.0%–<9.0%	40	16.88	20	17.39	20	16.39		
9.0%–16.2%	48	20.25	28	24.35	20	16.39		
Night-time voiding frequency (0–5)	M ± SD 1.69 ± 0.99		1.75 ± 0.87		1.63 ± 1.10		^†^ 0.91	0.364
0 episode	16	6.45	1	0.87	15	12.30		
1 episode	99	41.77	51	44.35	48	39.34		
2 epiosodes	77	32.49	43	37.39	34	27087		
≥3–5 episodes	45	18.99	20	17.39	25	20.49		
Poor sleepers (CPSQI>6)							2.15	0.143
Yes	139	58.65	73	63.48	66	54.10	(no vs. yes)	

Abbreviations: CPSQI, Chinese version of the Pittsburgh Sleep Quality Index; M, mean; SD, standard deviation; UI, urinary incontinence. Possible factors associated with urinary incontinence used in later independent *t* tests were age and body mass index, and Chi-squared tests included parity (<3 vs. ≥3 times), a history of vaginal delivery (no vs. yes), time since menopause (≤10 years vs. >10 years), chronic constipation (no vs. yes), smoking habit (no vs. yes), regular exercise (yes vs. no), caffeine consumption (no vs. yes), alcohol consumption (no vs. yes), hypertension (no vs. yes), time since diagnosis of diabetes (≤10 years vs. >10 years), and HbA1C (<7% vs. ≥7%). Chi-square test results of four variables (use of hormone replacement therapy, smoking habit, performing pelvic floor muscle exercise, and hysterectomy) were not shown because the sample size requirement were not satisfied. ^†^ *t* test. * *p* < 0.05, ** *p* < 0.01, *** *p* < 0.001.

**Table 2:** Symptom-related conditions in participants with urinary incontinence (*n* = 115).

Variable	*n*	%
Duration of urinary incontinence		
>1 month–<12 months	8	6.96
1–5 years	41	35.65
>5 years	66	57.39
Frequency of urinary incontinence		
A few times a month	57	49.56
A few times a week	32	27.83
Every day and/or night	26	22.61
Amount of urine leakage		
Drops	51	44.35
Small splashes	49	42.61
More	15	13.04
Sandvik’s severity index (range: 2–12, M ± SD = 4.99 ± 3.12)		
Slight (1–2)	46	40.00
Moderate (3–6)	38	33.04
Severe (8–9)	23	20.00
Very severe (12)	8	6.96
Type of urinary incontinence		
Stress urinary incontinence	62	53.91
Mixed urinary incontinence	49	42.61
Urge urinary incontinence	4	3.48

**Table 3:** Comparison of sleep quality and HRQoL by urinary incontinence condition (*n* = 237).

Sleep Quality (Mean ± SD)	Without UI (*n* = 122) ①	UI Severity		*F*	*p* Value	Post hoc: Scheffe or ^†^ Games–Howell
		Slight to Moderate (*n* = 84) ②	Severe to Very Severe (*n* = 31) ③			
The CPSQI global score	8.66 ± 4.27	7.92 ± 3.33	12.39 ± 4.17	14.920	<0.001	^†^ ③ > ① **, ③ > ② ***
Subjective overall sleep quality	1.73 ± 0.74	1.60 ± 0.78	2.32 ± 0.79	10.571	<0.001	③ > ① **, ③ > ② ***
Sleep latency	1.61 ± 1.10	1.51 ± 0.92	1.84 ± 0.82	1.189	0.306	
Sleep duration	1.80 ± 0.86	1.49 ± 1.02	1.65 ± 0.91	2.726	0.068	
Habitual sleep efficiency	1.22 ± 1.06	1.02 ± 0.99	1.42 ± 1.09	1.868	0.157	
Sleep disturbances	1.07 ± 0.33	1.11 ± 0.31	1.71 ± 0.53	41.810	<0.001	^†^ ③ > ① ***, ③ > ② ***
Use of sleeping medication	0.61 ± 1.10	0.38 ± 0.86	1.23 ± 1.25	7.384	0.001	^†^ ③ > ① *, ③ > ② **
Daytime dysfunction	0.62 ± 0.78	0.81 ± 0.72	2.23 ± 0.92	52.797	<0.001	^†^ ③ > ① ***, ③ > ② ***
HRQoL						
Physical component summary	47.24 ± 7.84	47.17 ± 7.71	46.66 ± 7.98	0.068	0.934	
Mental component summary	54.42 ± 5.53	52.70 ± 7.21	49.69 ± 6.49	7.376	0.001	③ < ① **

Abbreviations: CPSQI, Chinese version of the Pittsburgh Sleep Quality Index; HRQoL, health-related quality of life; SD, standard deviation; UI, urinary incontinence. ^†^ Games–Howell. * *p* < 0.05, ** *p* < 0.01, *** *p* < 0.001.

**Table 4:** Multivariate linear regression of sleep quality in participants with urinary incontinence (*n* = 115).

	Sleep Quality (the CPSQI Global Score)						
	Unstandardized			Standardized	*t*	*p*	VIF
	B	SE	95% CI	Β			
Urinary incontinence severity	0.371	0.139	0.095, 0.647	0.284	2.662	0.009	1.693
Nighttime voiding frequency	1.539	0.413	0.721, 2.358	0.327	3.728	<0.001	1.149
Age	−0.026	0.071	−0.165, 0.114	−0.035	−0.362	0.718	1.403
Body mass index	0.065	0.087	−0.107, 0.238	0.065	0.749	0.455	1.113
*R*^2^ = 0.261 *R*^2^*_adj_* = 0.234			*F* = 9.724	*p* < 0.001			

Abbreviations: CI, confidence interval; CPSQI, Chinese version of the Pittsburgh Sleep Quality Index; SE, standard error; VIF, variance inflation factor.

## Data Availability

Data are available on request due to privacy and ethical restrictions.
